# The combined influences of local heat application and resistance exercise on the acute mRNA response of skeletal muscle

**DOI:** 10.3389/fphys.2024.1473241

**Published:** 2024-10-21

**Authors:** Mark L. McGlynn, Alejandro M. Rosales, Christopher W. Collins, Dustin R. Slivka

**Affiliations:** ^1^ School of Health and Kinesiology, University of Nebraska at Omaha, Omaha, NE, United States; ^2^ School of Integrative Physiology and Athletic Training, University of Montana, Missoula, MT, United States

**Keywords:** myogenic, proteolytic, gene expression, myostatin, myogenin, *myogenic differentiation factor 1*

## Abstract

**Introduction:**

The development and maintenance of the skeletal muscle is crucial for the support of daily function. Heat, when applied locally, has shown substantial promise in the maintenance of the muscle. The purpose of this study was to determine the combined effects of local heat application and acute resistance exercise on gene expression associated with the human muscle growth program.

**Materials and methods:**

Participants (n = 12, 26 ± 7 years, 1.77 ± 0.07 m, 79.6 ± 15.4 kg, and 16.1 ± 11.6 %BF) completed an acute bilateral bout of resistance exercise consisting of leg press (11 ± 2 reps; 170 ± 37 kg) and leg extension (11 ± 1 reps; 58 ± 18 kg). Participants wore a thermal wrap containing circulating fluid (40°C, exercise + heat; EX + HT) during the entire experimental period and 4 h post-exercise, while the other leg served as an exercise-only (EX) control. Biopsies of the *vastus lateralis* were collected (Pre, Post, and 4hPost) for gene expression analyses.

**Results:**

Intramuscular temperatures increased (Post, +2.2°C ± 0.7°C, and *p* < 0.001; 4hPost, +2.5°C ± 0.6°C, and *p* < 0.001) and were greater in the EX + HT leg post-exercise (+0.35°C ± 0.3°C, and *p* = 0.005) and after 4hPost (+2.1°C ± 0.8°C and *p* < 0.001). *MYO-D1* mRNA was greater in the EX + HT leg vs. the EX (fold change = 2.74 ± 0.42 vs. 1.70 ± 0.28, *p* = 0.037). No other genes demonstrated temperature sensitivity when comparing both legs (*p* > 0.05). mRNA associated with the negative regulator, *myostatin* (*MSTN*), decreased post-exercise (*p* = 0.001) and after 4 h (*p* = 0.001). mRNA associated with proteolysis decreased post-exercise (*FBXO32*, *p* = 0.001; *FOXO3a*, *p* = 0.001) and after 4 h (*FBXO32, p* = 0.001; *FOXO3a*, *p* = 0.027).

**Conclusion:**

The elevated transcription of the *myogenic differentiation factor 1* (*MYO-D1*) after exercise in the heated condition may provide a mechanism by which muscle growth could be enhanced.

## 1 Introduction

The development and maintenance of the skeletal muscle is crucial for the support of daily function (Powers et al., 2016). Those with limited exercise capacities could benefit from supplementary support to enhance the potential for muscle growth. Local heat applied to the skin has shown promise as a non-invasive intervention that can promote a proangiogenic environment and potentially restrain the decline in mitochondrial function and muscle atrophy (Hafen et al., 2019; Hafen et al., 2018; Kim et al., 2020; Petrie et al., 2016; Sabapathy et al., 2021). Some have encouraged the use of local heat application to reduce time lost due to injury and optimize acute exercise muscle adaptations in the pursuit of improved performance (Hyldahl and Peake, 2020; Racinais et al., 2017). Additionally, passive (without exercise) heat application interventions have demonstrated improved strength, which the authors attribute to skeletal muscle hypertrophy (Goto et al., 2011). Although the mechanisms for these gains are unclear, muscle growth is dependent upon the initial transcriptional signals and mTOR upstream signaling as a function of mechanical stretch and nutrient demand (Hornberger and Chien, 2006). Therefore, by combining local heat application with the most potent muscle growth stimulator, resistance exercise (Welle et al., 1999), there may be an additive effect through which we can observe via quantifiable skeletal muscle transcriptional signals.

The combination of local heat + resistance exercise may enhance the benefit of an acute resistance exercise session. In theory, heat should reduce the activation energy requirements and increase the speed of biochemical reactions, leading to an enhanced muscle growth signal; however, homeotherms must employ thermoregulatory actions to prevent overheating (Geneva et al., 2019). These actions take advantage of convection by dilating the peripheral vasculature to deliver heated blood away from working muscle toward the cooler skin (via evaporated sweat). Cooled blood in the periphery then returns to the trunk of the body and contributes to the cooling of overheating organs, in this case, the heat-generating skeletal muscle (Beker et al., 2018). However, when deploying a local heat intervention to the skin of the exercising muscle, the effectiveness of these countermeasures fails and intramuscular temperature rises. Independent of exercise, some speculate that this state of elevated temperature and shift in cellular substrate utilization (toward a reliance on glucose) is a more suitable environment for muscle repair (Goto et al., 2011; Hafen et al., 2019; Hafen et al., 2018; Kim et al., 2020; Petrie et al., 2016; Sabapathy et al., 2021; Slivka et al., 2012); however, others were unsuccessful in observing any heat-related benefits (Labidi et al., 2021; McGlynn et al., 2023a).

The goal of designing a chronic resistance training program that utilizes heat stress has shown some promise with evidence of increased strength (Bartolomé et al., 2021; Goto et al., 2007; Nakamura et al., 2019) and increased muscle thickness (Goto et al., 2007; Nakamura et al., 2019), while others have observed no added benefit (Miles et al., 2019; Stadnyk et al., 2018). These differences can be partially explained by major differences in methodology, including muscle groups (biceps brachii, triceps brachii, and quadriceps group), types of measurement (muscle cross-sectional area, muscle thickness, and handgrip testing), heat applications (heat sheet, heating pad, and whole-body temperature-controlled chamber), and the length of resistance training interventions (3–12 weeks). Hence there is a need for a highly controlled, acute exercise research design to measure the earliest of molecular signals leading to future potential muscle growth.

Generally, initial muscle restructuring signals consist of nuclear production of mRNA (transcription) that code for specific proteins (translation) associated with downstream growth (myogenic) and breakdown (proteolytic) of the skeletal muscle. More importantly, local heat application evidence suggests that genes for these muscle restructuring processes are acutely sensitive to the application of local temperature (Hafen et al., 2018; Hafen et al., 2019; Kakigi et al., 2011; McGlynn et al., 2023a; Zak et al., 2017; Zak et al., 2018) and generally seem to peak within 4–8 h post-exercise (Yang et al., 2005). Therefore, by examining the influence of resistance exercise on the initial muscle restructuring signals (i.e., relative change in mRNA) found within a purported beneficial muscle repair molecular environment, one can quantify the combined responses of resistance exercise and local heat application. Thus, the purpose of this study was to quantify the genetic transcriptional changes associated with the muscle growth program after an acute session of bilateral resistance exercise between the locally heated muscle and non-heated muscle. Genes of interest included myogenic regulatory factor genes (*myogenic differentiation 1*, *MYO-D1*; *myogenin*, *MYO-G*; *myogenic factor 5*, *MFY5*; and *myogenic factor 6*, *MYF6*), a DNA binding enhancer factor gene (*myocyte enhancer factor 2a*, *MEF2a*), a negative regulator of myogenesis (*myostatin*, *MSTN*), and and ribosomal-associated genes (*ribosomal protein S3*, *RPS3* and *ribosomal protein L3-*like, *RPL-3L*). The genes of interest related to proteolysis were *F-box protein 32* (*FBXO32* (*Atrogin-1*)), *forkhead box O3* (*FOXO3a*), and *E3 ubiquitin ligase* (*TRIM63* (*MURF-1*)).

## 2 Materials and methods

### 2.1 Subjects

Thirteen individuals between the ages of 19 and 45 were recruited to participate in two visits to the exercise physiology laboratory: for initial an experimental testing. Participants were informed of experimental procedures and risks of participation prior to completing an approved consent document, medical history questionnaire (PAR-Q+) (Warburton et al., 2018), and COVID-19 status. All procedures were approved by an institutional review board and conducted in accordance with the guidelines of the Declaration of Helsinki. Based on these answered questionnaires, all subjects were considered to be of low risk for cardiovascular events (free of any positive risk factors), apparently healthy and physically active (Warburton et al., 2018), and free from any temperature-sensitive disorders (e.g., Raynaud’s syndrome), but currently not engaged in any formal resistance training program. One subject withdrew during testing due to emesis of the standard meal, leaving 12 subjects (n = 10 males and n = 2 females; 26.3 ± 7.1 years of age) in all analyses.

### 2.2 Initial testing

Subjects were instructed to arrive ready to provide maximal resistance exercise effort and must have fasted for at least 4 h. Initial testing included determination of height (1.77 ± 0.07 m (Seca stadiometer; Hamburg, Germany), weight (79.6 ± 15.4 kg) (Befour digital scale; Saukville, WI, United States), and body composition via bioelectrical impedance analysis (16.1% ± 11.6% body fat) (InBody body composition analyzer S10; InBody, United States, Cerritos, CA). The repetition maximum testing consisted of a subject-determined warm-up protocol involving light (1 set of 10 repetitions), medium (1 set up 5 repetitions), and heavy (1 set of 1 repetition) loads, with 1–2 min of rest between each set. Based on this performance, subjects were then instructed to execute as many repetitions as possible using the estimated 12-RM weight for both leg press and leg extension machines, and weights were adjusted accordingly for the next experimental visit.

### 2.3 Experimental visit

Participants arrived at the exercise physiology laboratory for the experimental visit having been instructed to fast overnight and avoid strenuous exercise and avoid consumption of alcohol, tobacco, or other recreational drugs for the previous 24 h. The combined thermal wraps, hip/groin (left #590604, right #590602) and articulated knee (#590106), allowed heating circulating fluid (Med4 Elite, Game Ready, CoolSystems Inc., Concord, CA, United States) to be applied to the entire experimental thigh following randomization and counterbalancing based on the temperature condition and biopsy order. Each subject served as their own control by simultaneously receiving the hot local application intervention on the experimental limb (fluid circulating 40°C; EX+HT), and the other leg served as an exercise-only (EX) control. The collection of muscle samples and recording of physiological (skin and intramuscular) temperatures occurred prior to any treatment (Pre), immediately post-exercise (Post), and after 4 h post-exercise (4hPost) with the continuous application of local heat treatment to the *vastus lateralis*. After collection of the Pre-muscle samples and recording of physiological temperatures from each leg, thermal wraps were applied to the *vastus lateralis* area for a 30-min pre-heating session immediately prior to the resistance exercise session. Upon initiating the 30-min preheating, subjects were fed a standard meal to ensure nutritional support for exercise and recovery (623 ± 60 Kcal; 84.2 ± 12.1 g of carbohydrate, 27.9 ± 0.1 g of protein, and 21.3 ± 1.5 g of fat). Subjects completed leg press and leg extension exercises aimed at hypertrophy (Baechle and Earle, 2008), executing 4 sets of 8–12 repetitions to exhaustion with continued weight adjustments with fatigue (see Table 1). Subjects were given 2 min of rest between sets and 5 min of rest between exercises, totaling 26 ± 1 min to complete the entire resistance exercise session designed to activate the *vastus lateralis*. Local heat application was applied continuously (totaling 317 ± 6 min), during rest and exercise, with necessary removal during participant transfers (<1 min), muscle biopsies (∼10 min each), and bathroom breaks (1–2 restroom breaks at about 5 min), totaling ∼30–40 min of potential open-air cooling over the 5+ hours.

**TABLE 1:** Resistance exercise.

	Set 1	Set 2	Set 3	Set 4
Leg press				
Repetitions	11 ± 2	11 ± 2	11 ± 2	11 ± 2
Load (kg)	174 ± 36	173 ± 36	169 ± 36	164 ± 40
Volume Load/Set (kg)	1856 ± 599	1854 ± 516	1812 ± 596	1816 ± 543
Leg extension				
Repetitions	10 ± 2	10 ± 2	11 ± 1	12 ± 1
Load (kg)	69 ± 20	62 ± 19	54 ± 15	48 ± 17
Volume Load/Set (kg)	700 ± 262	625 ± 183	597 ± 202	558 ± 184

Note: local heat + bilateral resistance exercise was executed until exhaustion; mean ± SD.

### 2.4 Physiological temperatures and biopsies

Skin temperature was measured immediately after the wraps were removed by using a laser thermometer (Fluke 62 Max+, Fluke Corp., Everett, WA, United States) aimed at the approximate biopsy location and recorded. A measure of 3 mL of 1% lidocaine was injected into the subcutaneous tissue and fascia adjacent to the biopsy site to numb the area. Betadine, a topical antiseptic bactericide, was spread on the *vastus lateralis* to sterilize the skin. Intramuscular temperature was measured at a depth of ∼4 cm by using a hypodermic thermocouple (26 gauge; MT-26/4HT Physitemp Instruments LLC, Clifton, NJ, United States) and recorded using a data logger (Extech Instruments, Nashua, NH, United States). A 5-mm percutaneous biopsy needle was inserted into the belly of the muscle to collect the muscle sample (∼80 mg) with the aid of suction (Bergstrom, 1962). These procedures were then repeated on the other limb with limited delay (11 ± 1 min). After removal of excess blood and connective tissue, samples were immersed in Allprotect (Allprotect, QIAGEN, Hilden, North Rhine-Westphalia, Germany) and stored at 4°C overnight, followed by storage at −30°C for later analyses.

### 2.5 Muscle processing and analyses

Real-time reverse transcription quantitative polymerase chain reaction (RT-qPCR) was utilized to analyze the mRNA content in the muscle samples. Skeletal muscle tissue (15.2 ± 2.9 mg) was homogenized in 500 µL of TRIzol (Invitrogen, Carlsbad, CA, United States) by using a handheld homogenizer (Homogenizer 150, Fisher Scientific, Waltham, MA, United States). Samples were incubated for 5 min at room temperature, and then 100 µL of chloroform was added and shaken by hand for 15 s. After another incubation at room temperature (2–3 min), the samples were centrifuged at 12,000 x g for 15 min at 4°C, and the aqueous layer was transferred to a fresh tube. A measure of 250 μL of isopropyl alcohol was added and mixed before another incubation, this time overnight at −20°C. The next day, samples were centrifuged immediately at 12,000 x g for 10 min at 4°C. The mRNA was washed by removing the supernatant, adding at least 500 μL of 75% ethanol, and then spun again for 5 min at 10,000 x g. The resulting mRNA pellet was dried and resolubilized in 30 µL of RNA storage solution (THE RNA Storage Solution, Invitrogen AM7000) and quantified (276.4 ± 83.6 ng·uL^−1^) and assessed for quality (RIN = 8.6 ± 0.5) using a fluorometer (Qubit 4, Invitrogen, Thermo Fisher). RNA was converted to complementary DNA (cDNA) using the SuperScript IV First-Strand Synthesis Kit with ezDNase (Invitrogen #18091050) according to the manufacturer’s instructions and diluted to 1.5 ng·uL^−1^. RT-qPCR reactions were run in 20-μL reactions containing 1 μL of the probe and primer mix (see Supplementary Appendix SA; gene primers and probe sequences), 10 μL of PrimeTime Gene Expression Master Mix (Integrated DNA Technologies, San Diego, CA, United States), 5 μL of deionized water, and 4 μL of template cDNA. Finally, samples were prepared for thermocycling (Agilent Technologies, AriaMx real-time PCR detection system, Santa Clara, CA, US) at 1 cycle of 95°C for 3 min, 40 cycles of 95°C for 5 s each, and 60°C for 10 s.

### 2.6 Gene expression analyses

The 2^−ΔΔCT^ method (Livak and Schmittgen, 2001) was used for the quantification and normalization of mRNA for genes of interest relative to the geometric mean of four reference genes (*β-actin*; *ACTB*, *β2-microglobulin*, *B2M*; *ribosomal protein S18*, *RPS18*; and *glyceraldehyde-3 phosphate dehydrogenase*, *GAPDH*). The geometric mean of the reference genes was used as the stable reference point for each participant. Stability was analyzed within individual subjects using the NormFinder algorithm (Andersen et al., 2004) to ensure that each subject was represented as an independent observation. If a reference gene for a given individual had a stability value of >0.15, it was removed from the analysis, and the geometric mean of the remaining reference genes was applied to the 2^−ΔΔCT^ method. Although there are many related genes involved in the complex action of muscle growth and breakdown, specific genes were chosen based upon their relation with myogenic and proteolytic potential and previous methodological effectiveness in measurement (McGlynn et al., 2023a; McGlynn et al., 2023b). The genes of interest related to myogenesis were *myogenic differentiation 1* (*MYO-D1*), *myogenin* (*MYO-G*), the negative regulator *myostatin* (*MSTN*), *myogenic factor 5* (*MFY5*), *myogenic factor 6* (*MYF6*), *myocyte enhancer factor 2a* (*MEF2a*), *ribosomal protein S3* (*RPS3*), and *ribosomal protein L3-like* (*RPL3L*). The genes of interest related to proteolysis were *F-box protein 32* (*FBXO32* (*Atrogin-1*)), *forkhead box O3* (*FOXO3a*), and *E3 ubiquitin ligase* (*TRIM63* (*MURF1*)).

### 2.7 Statistical analyses

A repeated measure two-way ANOVA, time (Pre, Post, and 4hPost) x temperature (EX vs. HT+EX) was used to compare dependent variables. If the F-ratio was found to be significant, then a Fisher’s protected least significant difference *post hoc* test was performed to evaluate where differences occurred. The probability of type I error less than 5% was considered significant (*p* < 0.05). All statistical data were analyzed using the Statistical Package for Social Sciences software (SPSS) (v. 27; IBM Corp., Armonk, NY). 2^−ΔΔCT^ mRNA analyses do not follow a normal distribution; therefore, the appropriate statistical analyses were performed on log-transformed data. If the assumption of sphericity was violated, the appropriate (Greenhouse–Geisser) correction was applied. Previous data on exercise and local heat application from our laboratory (Zak et al., 2018) have demonstrated that at 80% power, a sample size of n = 12 is necessary to observe a treatment effect.

## 3 Results

### 3.1 Thermoregulation

**FIGURE 1:**
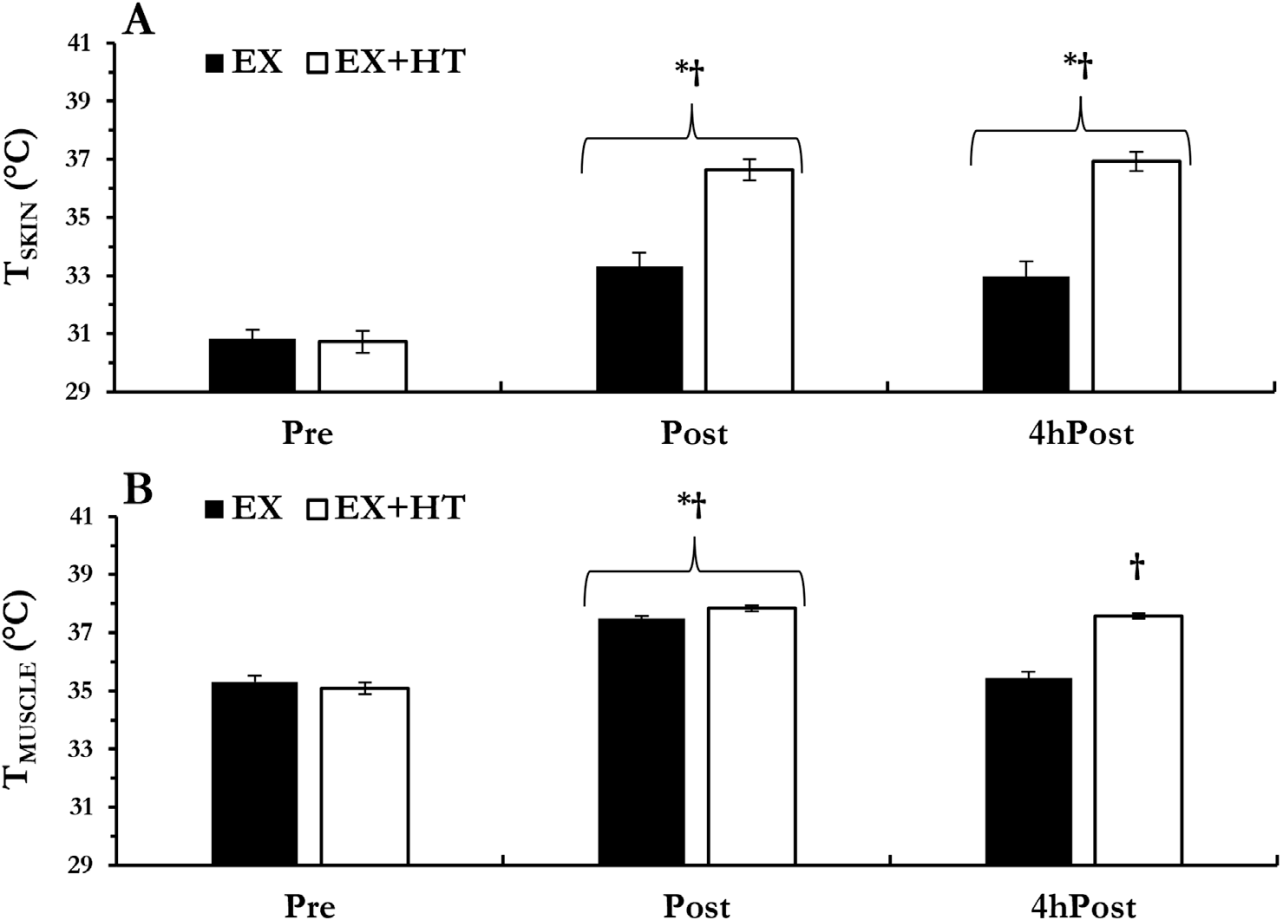
Temperatures: skin **(A)** and intramuscular **(B)** temperatures were documented prior to biopsy collection, immediately post-exercise (Post), and 4 h post-exercise (4hPost) in both the exercise-only leg (EX) and the exercise + heat leg (EX + HT). Data are presented as mean ± SEM. * different from pre; † EX + HT > EX.

Skin temperature increased from Pre regardless of temperature conditions (Post, *p* < 0.001 and 4hPost, *p* = 0.002) and was greater in the EX+HT leg vs. the EX leg at Post (*p* < 0.001) and 4hPost (*p* < 0.001). Intramuscular temperature increased from Pre regardless of temperature conditions (Post, *p* < 0.001) and was greater in the EX+HT leg vs. the EX leg at Post (*p* = 0.005). As expected, the EX intramuscular temperature returned to baseline 4hPost (*p* = 0.523) but remained elevated in EX+HT 4hPost (*p* < 0.001; see Figure 1).

### 3.2 Myogenic mRNA

Relative fold changes in immediately post-exercise myogenic genes of interest were normalized to Pre (fold change = 1.0) for the EX and the EX+HT and were compared (see Table 2). No temperature condition differences were observed immediately post-exercise (*p* > 0.05), so legs were combined. MYO-D1 mRNA increased from Pre to Post (*p* < 0.001, ηp2 = 0.446, observed power = 0.999). All other post-exercise mRNA signals associated with proteins responsible for myogenic processes went unchanged (*MYO-G*, *p* = 0.435; *MSTN*, *p* = 0.752; *MYF5*, *p* = 0.059; *MYF6*, *p* = 0.160; *MEF2a*, *p* = 0.722; *RPS3*, *p* = 0.226; and *RPL3-L*, *p* = 0.236).

**TABLE 2:** Relative fold change in genes of interest normalized to pre-exercise (fold change = 1.0).

	Immediately post-exercise			4 h post-exercise		
	EX	EX+HT	Combined	EX	EX+HT	Combined
Myogenic mRNA						
*MYO-D1*	2.09 ± 0.35	1.88 ± 0.22	1.99 ± 0.20 *	1.70 ± 0.28	2.74 ± 0.42 †	2.22 ± 0.27 *
*MYO-G (myogenin)*	1.00 ± 0.06	0.98 ± 0.09	0.99 ± 0.05	2.69 ± 0.32	1.96 ± 0.40	2.32 ± 0.26 *
*MSTN*	0.97 ± 0.10	1.53 ± 0.39	1.25 ± 0.21	0.52 ± 0.13	0.87 ± 0.22	0.69 ± 0.13 *
*MYF5*	0.73 ± 0.08	1.14 ± 0.22	0.94 ± 0.12	0.65 ± 0.10	0.68 ± 0.11	0.66 ± 0.07 *
*MYF6*	1.40 ± 0.15	1.24 ± 0.16	1.32 ± 0.11	1.59 ± 0.27	1.19 ± 0.19	1.39 ± 0.17
*MEF2a*	1.17 ± 0.16	0.99 ± 0.18	1.08 ± 0.12	1.16 ± 0.12	0.90 ± 0.08	1.03 ± 0.08
*RPS3*	1.01 ± 0.04	0.93 ± 0.08	0.97 ± 0.04	1.13 ± 0.12	0.96 ± 0.06	1.04 ± 0.07
*RPL-3L*	0.94 ± 0.05	0.99 ± 0.13	0.97 ± 0.07	0.89 ± 0.07	1.02 ± 0.22	0.96 ± 0.11
Proteolytic mRNA						
*FBXO32 (Atrogin-1)*	1.12 ± 0.20	1.11 ± 0.21	1.11 ± 0.14	0.57 ± 0.16	0.92 ± 0.29	0.74 ± 0.17 *
*FOXO3a*	1.33 ± 0.16	1.20 ± 0.20	1.27 ± 0.13	0.93 ± 0.14	0.81 ± 0.15	0.87 ± 0.10 *
*TRIM63 (MURF-1)*	1.42 ± 0.18	1.33 ± 0.23	1.37 ± 0.14 *	1.99 ± 0.47	1.57 ± 0.29	1.78 ± 0.27 *

Note: Relative fold change in genes of interest normalized to Pre-exercise (fold change = 1.0) for the exercise-only leg (EX) and the exercise + heat leg (EX+HT). Myogenic genes of interest were *myogenic differentiation 1 (MYO-D1)*, *myogenin (MYO-G)*, *myostatin (MSTN)*, *myogenic factor 5 (MFY5)*, *myogenic factor 6 (MYF6)*, *myocyte enhancer factor 2a (MEF2a)*, *ribosomal protein S3 (RPS3)*, *and ribosomal protein L3-like (RPL3L)*. Proteolytic genes of interest were *F-box protein 32 (Atrogin-1)*, *forkhead box O3 (FOXO3a)*, and *E3 ubiquitin ligase (MURF-1*, *aka TRIM63)*. Reference genes were *ACTB*, *B2M*, *RPS18*, and *GAPDH*; see Supplementary Appendix SA for gene primers and probes sequences. Data are mean ± SEM. *p < 0.05 is different from Pre. †*p* < 0.05 EX+HT > EX.

Relative fold changes in 4hPost myogenic genes of interest were normalized to Pre (fold change = 1.0) for the EX and the EX+HT and were compared. The only temperature condition differences occurred 4hPost in *MYO-D1* mRNA, which increased in EX + HT leg vs. EX leg (interaction, *p* = 0.037, ηp2 = 0.139, and observed power = 0.629). No other myogenic-related genes were influenced 4hPost by the temperature condition (*MYO-G*, *p* = 0.111; *MSTN*, *p* = 0.166; *MYF5*, *p* = 0.305; *MYF6*, *p* = 0.102; *MEF2a*, *p* = 0.161; *RPS3*, *p* = 0.156; and *RPL3-L*, *p* = 0.887). No other temperature condition differences were observed in 4hPost (*p* > 0.05), so legs were combined. After combining legs, *MYO-G* mRNA was elevated in 4hPost (*p* < 0.001) and trended toward an interaction (reduced mRNA within EX + HT; *p* = 0.071, ηp2 = 0.123, with limited observed power = 0.488). Interestingly, *MYF5* (*p* < 0.001, ηp2 = 0.331, and observed power = 0.986) and *MSTN* (*p* < 0.001, ηp2 = 0.392, and observed power = 0.989) mRNA were less in 4hPost. All other myogenic mRNAs went unchanged in 4hPost (*MYF6*, *p* = 0.153; *MEF2a*, *p* = 0.657; *RPS3*, *p* = 0.975; and *RPL3-L*, *p* = 0.108; see Table 2).

### 3.3 Proteolytic mRNA

Relative fold changes in Post- and 4hPost-exercise proteolytic genes of interest were normalized to Pre (fold change = 1.0) for the EX and the EX + HT and were compared (see Table 2). No temperature condition differences were observed for the proteolytic mRNA (*p* > 0.05), so legs were combined. *TRIM63* (aka *MURF-1*) mRNA increased due to exercise from Pre to Post (*p* = 0.011, ηp2 = 0.186, and observed power = 0.789), and from Pre to 4hPost (*p* = 0.013). *FBXO32* (aka *Atrogin-1*) mRNA decreased due to exercise, but only after 4hPost (4hPost, *p* < 0.001), not immediately post exercise (Post, *p* = 0.671). *FOXO3a* mRNA decreased due to exercise, but only after 4hPost (*p* = 0.027, ηp2 = 0.301, and observed power = 0.935), with a trend occurring immediately post exercise (Post, *p* = 0.088).

## 4 Discussion

It is of interest to thoroughly examine potential ergogenic aids that may enhance the benefit of an acute exercise session. The local application of heat has shown substantial promise in the development of an exercise intervention to benefit those with limited exercise capacities (Bartolomé et al., 2021; Fuchs et al., 2020; Goto et al., 2011; Goto et al., 2007; Hafen et al., 2019; Hafen et al., 2018; Kakigi et al., 2011; Kim et al., 2020; Nakamura et al., 2019; Petrie et al., 2016; Raue et al., 2006; Sabapathy et al., 2021). The aim of this investigation was to quantify transcriptional changes associated with the muscle growth program after an acute, heated session of bilateral resistance exercise. The main findings suggest that there is an acute gene expression benefit to the muscle growth program when the muscle is locally heated. The elevated expression of *myogenic differentiation factor 1* (*MYO-D1*) suggests potential for the synergistic effects of local heat application and resistance exercise to enhance skeletal muscle transcriptional signaling in the untrained, healthy participant. Specifically, a heightened differentiation signal initiates skeletal muscle satellite cells toward maturation and the potential for improved skeletal muscle maintenance or growth (Megeney et al., 1996). Whether these transcriptional upregulations lead to actual enhanced skeletal muscle growth cannot be determined here. More importantly, without this acute transcriptional potential, the likelihood of growth is slight. These findings add to the growing knowledge of skeletal muscle’s acute transcriptional response to the combined application of local heat and resistance exercise.

These results support previous heat-related applications intended to explore muscle growth signaling when combined with exercise. Although there are many local heat application investigations, few have explored the acute transcriptional response of skeletal muscle due to the combined effects of heat and resistance exercise (Zak et al., 2018). Previous work used a unilateral resistance exercise approach to compare local temperature applications but did not include a room temperature control (Zak et al., 2018). Nevertheless, similar changes were observed due to resistance exercise (i.e., elevated mRNA for *MYO-D1* and *MYO-G* with a concomitant depressed mRNA for *MYF5* and *MSTN*) after 4 h of local heated recovery. Muscle myotube research suggests a feedforward loop where *MYO-D1* and *MYO-G* enhance the expression of one another, yet the ultimate contributor to the differentiation of immature myoblasts seems to be *MYO-G* (Adhikari et al., 2021). This is noteworthy because even with methodological differences, both *MYO-D1* (within the current data) and *MYO-G* (Zak et al., 2018) exhibited elevated mRNA and thus demonstrated sensitivity to this heat + resistance exercise intervention. Finally, although not statistically significant, *MYO-G* within the EX+HT condition of the current project approaches an increase after 4 h (*p* = 0.069). Taken together, these data suggest that the genes associated with the differentiation of the *vastus lateralis* are sensitive to the application of local heat when combined with an acute resistance exercise session.

In addition, further comparisons can be made between the genes associated with proteolytic actions. Resistance exercise has consistently demonstrated its ability to downgrade the gene associated with the negative regulator for muscle growth, *myostatin*, as early as 1 h and lasting up to 1 day post-exercise (Louis et al., 2007; Zak et al., 2018). The current investigation’s mRNA for *myostatin* (*MSTN*) supports the results of the previous studies, suggesting its mRNA is not sensitive to this combined EX+HT intervention. Other genes associated with the breakdown of muscle protein (*Atrogin-1*, *FOXO3a*, and *MURF-1*) seemed to follow the expected post-resistance exercise changes (decreased mRNA for *Atrogin-1* and *FOXO3a* and increases in *MURF-1*) and went unaffected by the addition of heat to the resistance exercise session (Louis et al., 2007; Zak et al., 2018). Taken together, there does not seem to be a detectable influence of heat over these essential proteolytic genes within this 4-h timeframe.

The current methods produced a higher temperature in the EX + HT leg (+0.35°C vs. EX muscle) immediately post-exercise and maintained an elevated temperature 4 h post-exercise (37.6°C), while the temperature of the non-heated leg returned to baseline (35.4°C). This applied approach to local temperature application was effective and executed with little effort. Although it was not measured, previous local heat application investigations have demonstrated minimal influences on core temperature-regulating countermeasures (Heesch et al., 2016). Other acute resistance exercise + heat investigations have produced mixed results including enhanced mTOR signaling within 1 h post-exercise using microwave radiation (Kakigi et al., 2011) and reduced post-exercise muscle damage due to pre-warming via water immersion (Sabapathy et al., 2021). These heat application interventions may be damaging to microwave radiation-sensitive signaling and cumbersome when attempting to simultaneously execute resistance exercise while underwater. Furthermore, the current methods applied heat stress to the thigh prior to exercise, during exercise, and during the 4 h of post-exercise recovery to maximize the intervention effect and match a previous non-exercise investigation (McGlynn et al., 2023a) that observed no transcriptional changes due to heat alone. It has been proposed that an elevated muscular temperature, independent of exercise, is a more suitable environment for muscle development based upon several local vasculature changes (i.e., increased blood flow and enhanced vasodilatory signals) (Goto et al., 2011; Hafen et al., 2019; Hafen et al., 2018; Petrie et al., 2016) and potential beneficial intracellular changes that have been documented by others, including potentiated heat shock proteins and favorable shifts in glucose utilization (Iguchi et al., 2012; Slivka et al., 2012). Although these methods cannot determine the most influential transcriptional timeframe, previous single-session investigations that used pre-warming (Kakigi et al., 2011; Sabapathy et al., 2021), heating during exercise (Kakigi et al., 2011), and heating only during the post-exercise recovery period (20 min) (Fuchs et al., 2020) yielded positive results for the development of the muscle growth program. However, the ultimate goal of translating these theorized favorable environments into long-term gains via chronic resistance training programs has produced inconclusive results (Bartolomé et al., 2021; Goto et al., 2007; Miles et al., 2019; Nakamura et al., 2019; Stadnyk et al., 2018). Nonetheless, these chronic training programs have produced some empirical evidence (i.e., increased strength and cross-sectional area of the biceps brachii after 10 weeks) (Goto et al., 2007), suggesting that there is potential for enhanced muscle growth. However, the exact temperature and exercise circumstances that would allow for such advantageous conditions are not clear and would be a complex task to produce during a weeks-long training investigation. Taken together, these methods may contribute to the use of local application of heat prior to exercise, during exercise, and throughout the acute post-exercise recovery (4 h) as a potential ergogenic aid for those who may benefit from an enhanced muscle growth program (i.e., untrained or those with limited exercise capacities).

To further put these transcriptional results into context, these data are nuclear markers associated with the potential for enhanced muscle growth. More importantly, for this potential to transform into an accelerated rate of muscle growth, this acute response must be repeated via a long-term training program. Furthermore, it would be an error to assume a similar acute transcriptional response over the course of a training program. Finally, even if the gene expression is elevated, there are additional steps beyond the transcription/translation via post-translational modifications that may further regulate the initial transcriptional signal. This is evident even within the current investigation as we observed enhanced myogenic gene expression paired with enhanced proteolytic expression (*TRIM63*, aka *MURF-1*). This demonstrates the complexity of the post-exercise muscle growth program response and the importance of balance between proteolytic and myogenic potential. Future research should continue to explore models that can better quantify the combined effects of temperature and resistance exercise on the human muscle growth program.

## 5 Conclusion

Many researchers have recommended the utilization of local heat application to maintain muscle growth because of its purported improved molecular environment for muscle repair. Here, we report elevated intramuscular temperatures immediately post-exercise and 4 h post-exercise due to the application of local heat to the *vastus lateralis* of one leg, which resulted in an enhanced transcription of *MYO-D1*. The thorough examination of these heat-focused muscle growth interventions should be a priority as it may provide a therapeutic target for those with limited exercise capacities.

## Data Availability

The data presented in the study are deposited in the publicly available repository figshare.com and can be accessed at https://doi.org/10.6084/m9.figshare.27180396.v1, McGlynn and Slivka, 2024.
