# Supplementary material for: The combined influences of local heat application and resistance exercise on the acute mRNA response of skeletal muscle
Source: Front Physiol. 2024 Oct 21;15:1473241. doi: 10.3389/fphys.2024.1473241 (PMC11532036; doi:10.3389/fphys.2024.1473241)
Supplement: Supplementary file 1 [file DataSheet1.docx]

**Supplementary Appendix SA. Gene primers and probes sequences**

|  | **Primer 1** | **Primer 2** | **Probe** |
| --- | --- | --- | --- |
| **Reference** |  |  |  |
| *ACTB* | CCTTGCACATGCCGGAG | ACAGAGCCTCGCCTTTG | TCATCCATGGTGAGCTGGCGG |
| *B2M* | ACCTCCATGATGCTGCTTAC | GGACTGGTCTTTCTATCTCTTGT | CCTGCCGTGTGAACCATGTGACT |
| *GAPDH* | TGTAGTTGAGGTCAATGAAGGG | ACATCGCTCAGACACCATG | AAGGTCGGAGTCAACGGATTTGGTC |
| *RSP18* | GTCAATGTCTGCTTTCCTCAAC | GTTCCAGCATATTTTGCGAGT | TCTTCGGCCCACACCCTTAATGG |
| **Myogenic** |  |  |  |
| *MYF5* | GGCATATACATTTGATACATCAGGAC | CACCTCCAACTGCTCTGATG | TGCTGTCAAAAGTACTGCTCTTTCTGGA |
| *MYF6* | CTACTCGAGGCTGACGAATC | CAGCTACAGACCCAAACAAGA | TGATAACGGCTAAGGAAGGAGGAGCA |
| *MYO-G* | AGAAGTAGTGGCATCTGTGG | GACAGCATCACAGTGGAAGA | ATGCCCGGCTTGGAAGACAATCT |
| *MYO-D1* | TGCTGGACAGGCAGTCTA | CTCCGACGGCATGATGG | TCGACACCGCCGCACTCTTC |
| *RPS3* | CCACTCTCCATGATGAACCG | CTAGAGGTCTGTGTGCCATTG | ACCATAGCAGGCCCTCCGC |
| *RPL3-L* | TGCTTTTTCCCGTCTGTGTC | ATCTTTGCAGAACACCTCAGT | TCTTGCTCTTGTGCCAGTCCTTGT |
| *MEF2a* | GTTTACAAATCCATTCCCCACTG | TCCTCAGAGACCACCAAGT | TCAACATCCCACCTGCATTGCC |
| *MSTN* | TCGTGATTCTGTTGAGTGCT | TGTAACCTTCCCAGGACCA | TCTTTTTGGTGTGTCTGTTACCTTGACCT |
| **Proteolytic** |  |  |  |
| *FBXO32* | TCAGCCTCTGCATGATGTTC | CAACAGACTGGACTTCTCAACT | CACTGACCTGCCTTTGTGCCTACA |
| *FOXO3a* | CCACCCTTGGCCTCTAAATAA | GGTAACAGGTATCAGGTTCTGG | ACATGACATGCGCTCTTGGGATCT |
| *TRIM63* | GCAACTCACTTTTCTTCTCATCC | TGCAGACCATCATCACTCAG | ACCTGGTGACTGTTCTCCTTGGTC |

**Note:** *β-actin (ACTB), β2-microglobulin (B2M), and glyceralde-hyde-3 phosphate dehydrogenase (GAPDH), ribosomal protein S18 (RPS18), myogenic factor (MFY5), myogenic factor 6 (MYF6), myogenin (MYO-G), myogenic differentiation 1 (MYO-D1), ribosomal protein S3 (RPS3), ribosomal protein L3-like (RPL3-L), myocyte enhancer factor 2a (MEF2a), myostatin (MSTN), F-box protein 32 (FBXO32, aka Atrogin-1), Forkhead Box O3 (FOXO3a), and E3 ubiquitin ligase (TRIM63, aka MURF-1).* Sequences purchased from PrimeTime RT-qPCR assays, Integrated DNA Technologies, San Diego, CA, USA.
